# Assessing Risk in Patients with Stable Coronary Disease: When Should We Intensify Care and Follow-Up? Results from a Meta-Analysis of Observational Studies of the COURAGE and FAME Era

**DOI:** 10.1155/2016/3769152

**Published:** 2016-04-27

**Authors:** Umberto Barbero, Fabrizio D'Ascenzo, Freek Nijhoff, Claudio Moretti, Giuseppe Biondi-Zoccai, Marco Mennuni, Davide Capodanno, Marco Lococo, Michael J. Lipinski, Fiorenzo Gaita

**Affiliations:** ^1^Division of Cardiology, University of Turin, Turin, Italy; ^2^Meta-Analysis and Evidence Based Medicine Training in Cardiology (METCARDIO), Rome, Italy; ^3^Department of Cardiology, University Medical Center Utrecht, Utrecht, Netherlands; ^4^Department of Medico-Surgical Sciences and Biotechnologies, Sapienza University of Rome, Latina, Italy; ^5^Department of Angiocardioneurology, IRCCS Neuromed, Pozzilli, Italy; ^6^Department of Interventional Cardiology, Istituto Clinico Humanitas, IRCCS, Rozzano, Italy; ^7^Cardiothoracovascular Department, Ferrarotto Hospital, University of Catania, Catania, Italy; ^8^MedStar Cardiovascular Research Network, MedStar Washington Hospital Center, Washington, DC, USA

## Abstract

*Background*. A large number of clinical and laboratory markers have been appraised to predict prognosis in patients with stable angina, but uncertainty remains regarding which variables are the best predictors of prognosis. Therefore, we performed a meta-analysis of studies in patients with stable angina to assess which variables predict prognosis.* Methods*. MEDLINE and PubMed were searched for eligible studies published up to 2015, reporting multivariate predictors of major adverse cardiac events (MACE, a composite endpoint of death, myocardial infarction, and revascularization) in patients with stable angina. Study features, patient characteristics, and prevalence and predictors of such events were abstracted and pooled with random-effect methods (95% CIs). Major adverse cardiovascular event (MACE) was the primary endpoint.* Results*. 42 studies (104,559 patients) were included. After a median follow-up of 57 months, cardiovascular events occurred in 7.8% of patients with MI in 6.2% of patients and need for repeat revascularization (both surgical and percutaneous) in 19.5% of patients. Male sex, reduced EF, diabetes, prior MI, and high C-reactive protein were the most powerful predictors of cardiovascular events.* Conclusions*. We show that simple and low-cost clinical features may help clinicians in identifying the most appropriate diagnostic and therapeutic approaches within the broad range of outpatients presenting with stable coronary artery disease.

## 1. Introduction

Coronary heart disease (CHD) is the leading cause of mortality and morbidity worldwide [1]. Prognostic assessment plays an important role in patient management in CHD as it enables the implementation of appropriate surveillance strategies. Although stable coronary artery disease (SCAD) represents the most common manifestation of ischemic heart disease, affecting up to 5% of the adult population over the age of 40 in developed countries [2, 3], it remains unclear which clinical features should be utilized by general physicians or cardiologists to determine the appropriate intensity of follow-up.

Although their role in risk stratification remains to be fully determined, a substantial number of biomarkers can provide additional information beyond that provided by traditional risk factors to predict cardiovascular events [4–7]. Additionally, the introduction of fractional flow reserve for guidance of percutaneous coronary intervention (PCI) was shown to reduce the need for urgent revascularization in patients with stable angina [8]. While angioguided PCI was not shown to reduce death and myocardial infarction (MI) in patients with stable angina [9], the ability to determine which patient characteristics have the greatest association with outcomes in patients with stable angina may help reduce major adverse cardiovascular events (MACE) through better treatment of these risk factors. Given the importance of reducing medical costs, identification of modifiable risk factors in patients with stable angina that may be easily treated and improve patient outcomes is critically important. Only a few studies have addressed these issues [2–5] and we are unaware of prior studies assessing which variables predict outcomes using meta-analysis in patients with stable angina. We therefore performed an international collaborative systematic review and meta-analysis to appraise the prevalence and predictors of cardiovascular events in patients with stable angina.

## 2. Methods

The main objective of this study was to identify multivariate predictors of MACE in patients diagnosed with SCAD using a meta-analytical approach. Current guidelines were followed during the course of the present research, in particular the recent Preferred Reporting Items for Systematic reviews and Meta-Analyses (PRISMA) amendment to the QUality Of Reporting Of Meta-analyses (QUOROM) statement and recommendations from the Cochrane Collaboration and Meta-analysis Of Observational Studies in Epidemiology (MOOSE) [10–15].

### 2.1. Search Strategy and Study Selection

MEDLINE, Cochrane Library, and BioMed Central were systematically screened in keeping with established methods [16] with highly specific terms “(cardiac OR myocard^∗^) AND (angina^∗^ OR ischemi^∗^) AND stable AND english[lang] AND ("2002" [pdat]  :  "2015" [pdat]) NOT (review[pt] OR editorial[pt] OR letter[pt])”. Two independent reviewers (FDA and UB) first analyzed selected citations at the title and/or abstract level, with disagreements resolved after consensus. If potentially pertinent, studies were then appraised as complete reports according to the following explicit selection criteria. Studies were included if (1) patients presenting with stable coronary disease or patients with stable angina or instrumental ischemia were investigated and if (2) predictors of cardiovascular events at follow-up identified through multivariate analysis were reported. All these criteria must be met for inclusion. Exclusion criteria were nonhuman studies, non-English language, and duplicate reporting (in which case the article reporting on the largest sample was retained).

### 2.2. Data Extraction

The same two investigators independently and in a blinded fashion tabulated the data of all studies qualifying for the meta-analysis and also contacted the corresponding authors for additional information [17]. Data collected included authorship, journal and year of publication, country of origin, baseline demographic and clinical features (e.g., risk factors, previous percutaneous or surgical revascularisation), and independent predictors of cardiovascular events. For each study that was included, both the frequencies and odds ratios (ORs) of the independent predictors were documented. MACE incidence was the primary endpoint.

### 2.3. Data Analysis and Synthesis

Continuous variables are reported as mean (SD) or median (range). Categorical variables are expressed as *n*/*N* (%). Statistical pooling was performed according to a random-effects model with generic inverse-variance weighting, computing risk estimates with 95% CIs, using RevMan 5 (the Cochrane Collaboration, the Nordic Cochrane Centre, and Copenhagen, Denmark). Fixed effect was also appraised and reported only if different results from randomness were found. We considered how well each study was adjusted for data collection by analyzing the kind of multivariate analysis used. Independent predictors were reported according to the number of studies in which they were evaluated and weighted according to their OR. Small study bias was appraised by graphical inspection of funnel plots. Standard hypothesis testing was set at the two-tailed 0.05 level.

## 3. Results

**Figure 1:**
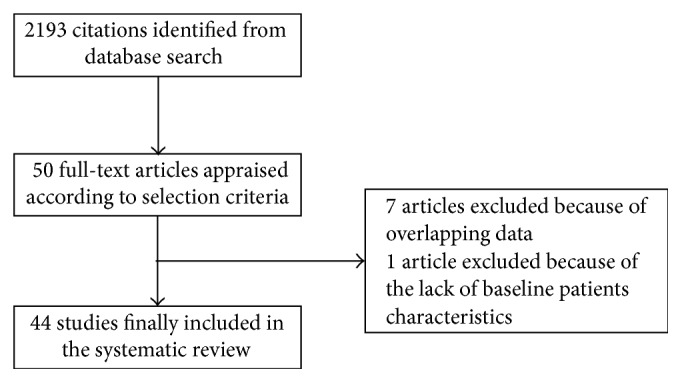
Search strategy results.

Search strategy results are presented in Figure 1. Forty-two reports were retained for meta-analysis, representing a total of 104,559 patients [18–59]. An overview of the included reports is given in Table 2. Stable coronary disease was defined uniformly in all studies as typical chest pain on exertion relieved by rest and/or sublingual nitrates, a positive ECG exercise test response (1 mm ST-segment depression), and/or reversible perfusion defects on myocardial perfusion in single-photon emission computed tomography (SPECT). In all patients, symptoms were stable for at least 2 months before study entry. Baseline patient features are reported in Table 1. Median follow-up was 57 months (IQR: 25–60).

**Figure 2:**
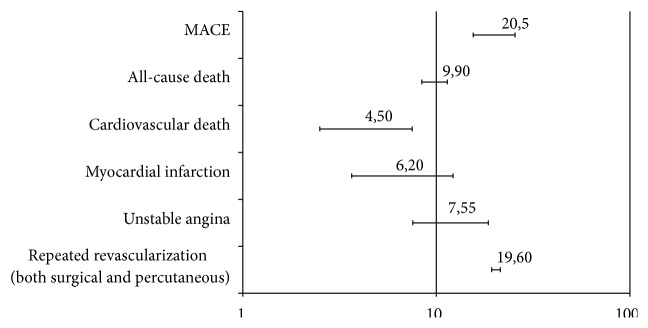
Incidence of adverse cardiovascular events after a follow-up of 57 months. MACE: major adverse cardiac events.

**Figure 3:**
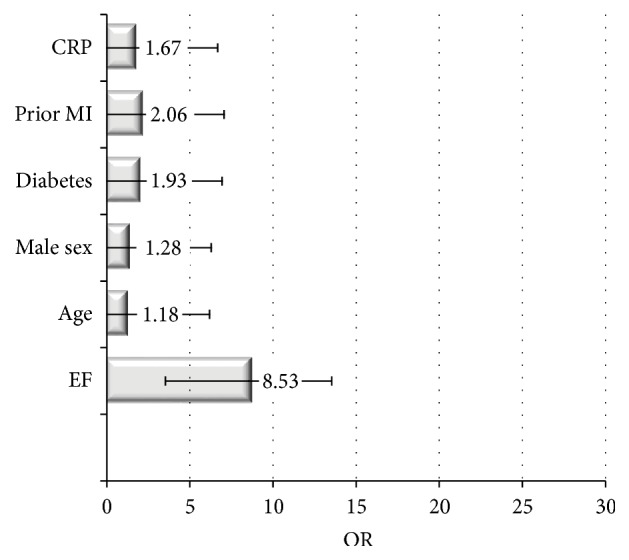
The most common predictors of subsequent CV events in stable angina patients. Data are reported as OR median value, with lower/upper limit confidence interval.

**Figure 4:**
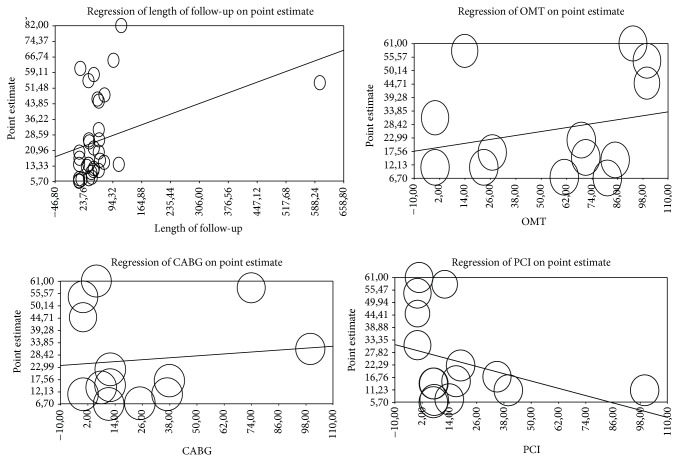
Effect of length of follow-up (beta 0.07; 0.03–0.09), of optimal medical therapy (0.02; 0.01–0.04), of CABG (0.04; −0.01–−0.06), and of PCI (0.03; 0.02–0.07) on CV events.

In SCAD patients, the overall incidence of cardiovascular events was 7.8% (95% CI: 5.89%–9.66%). The incidence of MACE was 20.5% (95% CI: 14.2–22.8), all-cause death was 9.9% (95% CI: 5.2–15), CV death was 4.5% (95% CI: 3–5.1), MI was 6.2% (95% CI: 4.2–9), and unstable angina was 7.6% (95% CI: 5–13). Furthermore, 19.5% of patients (95% CI: 14.25–24.95) required repetition of revascularization (either surgery or PCI). An overview of the incidence of cardiovascular events is presented in Figure 2. Metaregression analysis demonstrated that the left ventricular ejection fraction (LVEF) at clinical presentation (reported in 12% of studies) and a previous history of MI (reported in 14% of studies and thus regarding 34% of this subgroup of patients) were the most powerful predictors of new cardiovascular events (Figure 3). The other predictors were male sex (OR: 1.28, 95% CI: 1.13–3.4), diabetes mellitus (OR: 1.93, 95% CI: 1.1–11.2), and C-reactive protein (CRP, OR: 1.67, 95% CI: 1.21–6.41). Metaregression revealed no interaction between the index treatment patients received (PCI, CABG, or OMT) and the incidence of MACE during follow-up (Figure 4).

## 4. Discussion

This study demonstrates that (a) the incidence of cardiovascular events remains high in patients with stable coronary disease and (b) although we did not build a prediction model, we reported that simple, inexpensive, and readily available clinical and laboratory tests may be helpful in identifying patients at higher risk of developing subsequent events. Identification of high-risk individuals may enable initiation of timely and appropriate therapies to reduce cardiovascular symptoms and events. As recently stressed by the CALIBER study [60], risk stratification in SCAD patients is mandatory: a complete and useful prognostic model was derived from such study, but it is complex and needs an online risk calculator. The aim of our investigation is to define the most powerful predictors of events in SCAD patients in order to derive some strong points that each clinician could easily remember at the patient's bedside.

As a matter of fact, due to the great variability among patients with stable coronary disease, discerning when to intensify care and follow-up is still a trouble of physician experience and judgement. Our meta-analysis provides interesting information from an epidemiological point of view, revealing the most powerful predictors of cardiovascular events in patients with stable angina. This information is easily obtained and may provide a deeper insight into which patients with stable angina are really “stable.” Furthermore, this data may be used to help decide which patients should continue with optimal medical therapy or be referred for FFR-guided PCI.

LVEF at clinical presentation seems to be the most powerful index to recognize patients with higher risk of subsequent events. The prognostic significance of severely impaired left ventricular systolic function is well established in patients with heart failure, including those with coronary artery disease as the underlying aetiology [61, 62], and in those presenting with recent myocardial infarction [63]. Our analysis confirmed that, even in patients with stable coronary disease, left ventricular systolic function at baseline is a strong predictor of outcome, even after adjustment for other major prognostic factors such as age, blood pressure, and gender. The patients with reduced LV systolic function have a greater incidence of new overt heart failure, new myocardial infarction, and increased risk of CV death and stroke. This is potentially due, in part, to an increased propensity for arrhythmias, thrombosis, and systemic embolism. Additionally, these patients have a decreased ability to compensate hemodynamic changes in the setting of critical illness such as AMI and sepsis.

Previous studies in a broad spectrum of heart failure patients reported that the relationship between LVEF and outcome was curvilinear with evidence that an ejection fraction of less than 45% was associated with a worse prognosis [62]. In addition, subjects with an ejection fraction of less than 45% were more likely to have a history of higher blood pressure, suggesting that hypertension may have also contributed to left ventricular dysfunction [30]. The most recent guidelines on management of stable coronary disease reported that the strongest predictor of long-term survival is LV function, as demonstrated by the Coronary Artery Surgery Study (CASS) registry [64, 65]. Hence, a patient with an LVEF <50% is already at high risk for CV death (annual mortality 3%), even without accounting for additional event risk factors, such as the extent of ischemia. As the guidelines stressed, the clinician involved in the management of patients with a known or suspected diagnosis of stable angina should always be sure to obtain a resting echocardiogram.

*Myocardial Infarction*. In a previous analysis of the international REduction of Atherothrombosis for Continued Health (REACH) registry, the strongest predictor of future ischemic risk was a history of prior ischemic events, particularly in the prior year [66]. The concomitant presence of diabetes or multivessel disease in those patients identified an extremely high-risk population [66]. Similar results also emerged from our study, and this is probably due to the fact that previous MI selected a population of patients with angina with tendency to develop acute coronary syndromes. Moreover, this is a nonmodifiable risk factor. In the study by Gulliksson et al., based on the Swedish national MI registry with more than 1 million AMI events, the risk of a recurrent AMI was highly dependent on time from the previous event, a finding which may affect risk scoring. In addition, sex, age, and the number of prior MIs influence the general risk level [67]. Furthermore, several biomarkers reflecting inflammatory activity have been shown to be elevated for weeks to months following MI and this inflammatory response could aggravate existing atherosclerotic lesions by accelerating their growth and/or promoting plaque instability, fatal arrhythmia, or heart failure [68].

*Male Gender*. This is in agreement with data from the Framingham study [69] and more recent studies [70, 71], supporting the contention that women with stable angina pectoris have a much better prognosis than men.

The American College of Cardiology-National Cardiovascular Data Registry, an angiographic registry, confirmed that, in stable angina patients, the risk-adjusted OR for significant CAD (i.e., >70% coronary stenosis) was reduced for women compared with men (OR: 0.34, 95% CI: 0.33–0.34), with black women having the lowest risk-adjusted odds compared with other females. Nevertheless, in this registry, white women had a 1.34-fold (95% CI: 1.21–1.48) higher risk-adjusted OR for mortality than white men with stable angina. The Authors explain this higher in-hospital mortality for white women with the lower utilization of elective coronary revascularization, aspirin, and glycoprotein IIb/IIIa inhibitors when compared with men [61].

In the extended follow-up of the Angina Prognosis Study In Stockholm (APSIS), men were at a 3- to 4-fold increased risk of MACE compared with female patients of a similar age. Interestingly, the event rate was greatly increased in a small subgroup of diabetic female patients. Thus, identifying and treating diabetes appear to be particularly important among female patients with CV disease, as, in the absence of diabetes, the CV mortality of female patients with stable angina pectoris seems to be similar to that of the general population [35]. When evaluating stable coronary disease outpatients, it is important to recognize that men are at increased risk of adverse outcomes. However, it is important to acknowledge recent reports which found that women with extensive coronary calcification have higher mortality rates than men [71].

*Diabetes Mellitus*. It is a recognized risk factor for cardiovascular disease (CVD) and mortality. As previously mentioned, not only is diabetes associated with a worse cardiovascular prognosis, but also it can exacerbate and worsen other risk factors [72, 73]. Diabetes is known to worsen conventional risk factors among women [74–76]. In addition, a preferential treatment for men with diabetes, compared with diabetic women, has been described: diabetic men tend to receive more adequate pharmacological treatment for CVD prevention compared with their female counterparts [76–78]. In the APSIS study, patients with a fasting blood glucose concentration above 6.1 mmol/L (according to revised definitions of diabetes mellitus) carried a comparable risk for future events as those patients with a diagnosis of diabetes at baseline [35]. Thus, regardless of patient age or sex, patients with stable angina should be screened by checking glycated haemoglobin and therapy should be initiated if its level is elevated.

*CRP*. Several studies of single markers, including a study based on an earlier examination cycle of the Framingham Heart Study, have yet shown little improvement in the prediction of risk with the addition of CRP to conventional risk factors [79, 80]. Our study also found only a moderate association between high-sensitivity CRP and cardiovascular events, consistent with the previous meta-analysis by Danesh et al. [81]. Moreover, recent guidelines state that no recommendation can be made to routinely measure this parameter, reposing on a recent analysis of 83 studies which found substantial heterogeneity in reporting and publication bias, making the magnitude of any independent association between high-sensitivity CRP and prognosis among patients with stable CAD uncertain [82]. Nevertheless, CRP levels may still be useful to identify subgroups of patients that require a more stringent approach; for example, in patients who are at intermediate risk for cardiovascular events, increased CRP values may warrant a more aggressive modification of risk factors (lowering of serum cholesterol or blood pressure) [83].

When applying metaregression analysis on CV events with respect to the kind of therapy patients received (PCI, CABG, or OMT), we observed a trend of reduced events for PCI and a slight increase for CABG and OMT, but without statistical significance. This is in agreement with the current literature, as no evidence is available suggesting better outcomes for any of these therapies when compared to another. FFR-guided PCI, providing a functional rather than morphological assessment of stenosis, only promise some improvements in stable angina patients [84].

*Limitations*. B-type natriuretic peptide was not included in our analysis because of the limited number of studies evaluating this marker in stable angina patients. However, data suggest that it may have a stronger relation with overall cardiovascular risk than CRP, an observation that has been confirmed by studies assessing these biomarkers simultaneously in high-risk populations [54].

Smoking does not enter in our selection of risk factors, although it is a well-known and established risk factor. This is due to different classifications of smoking (former, current, or undetermined) in different studies. It is important to note that, in long-term registries such as the Angina Prognosis Study In Stockholm (APSIS), current smoking is an important and modifiable risk factor [35].

Finally, the term MACE, defined as “major adverse cardiac events” and commonly used as a composite endpoint in cardiovascular research, has no standard definition: including multiple types of clinical events of varying degrees of relatedness, validity, and utility of MACE could be questioned preferring separate endpoints that reflect both the safety and effectiveness of various treatment approaches [85]; furthermore, in many articles, not all the components of MACE were investigated.

## 5. Conclusion

Our study demonstrates that patients with stable angina pectoris at increased risk may be easily identified by assessing several common and important risk factors. In patients with increased risk, aggressive risk factor modification and medical therapy should be instituted and FFR-guided PCI considered if symptoms of angina are present. We found that ejection fraction, sex, diabetes mellitus, previous MI, and CRP independently predicted an increased risk during a cumulative five-year follow-up. Thus, these risk factors may identify patients in need of further angiographic investigation while a conservative strategy may be safely adopted in low-risk patients.

## Figures and Tables

**Table 1:** Baseline characteristics of patients (*n* = 104559) of the 44 studies included. The first column shows variables, the second one shows the values expressed as mean percentage ± SD, and the third one shows the number of studies reporting each variable.

Variable	Value	*n* of studies
Age (years)	63.5 ± 4.1	42
Female gender (%)	26.6 ± 8.6	42
Diabetes mellitus (%)	23.2 ± 12.8	42
Hypertension (%)	58.7 ± 16.4	42
Hyperlipidemia (%)	63.8 ± 13.4	24
Smoking (%)	33.9 ± 19.1	42
Family history (%)	42.4 ± 11.8	16
Alcohol use (%)	29.5 ± 0.5	3
Physically inactive (%)	51.5 ± 14.8	3
Prior stroke (%)	9.05 ± 4.08	6
Prior AMI (%)	38.8 ± 18.8	35
Prior CABG (%)	11.8 ± 12.1	22
Prior PCI (%)	18.2 ± 19.6	21

**Table 2:** List of included studies with the number of patients involved and the type of treatment (PCI: percutaneous coronary angioplasty; CABG: coronary artery bypass graft; MT: medical therapy; ND: not reported data).

Study ID	Number of patients	Treatment
Aguilar et al., 2006 [18]	3319	ND
Arroyo-Espliguero et al., 2009 [19]	790	PCI, MT
Avanzas et al., 2005 [20]	297	PCI, MT
Bhatt et al., 2010 [21]	45227	ND
Borges et al., 2010, CABG [22]	136	CABG
Borges et al., 2010, MT [22]	110	MT
Borges et al., 2010, PCI [22]	146	PCI
Breeman et al., 2006 [23]	2928	PCI, MT, CABG
Carpeggiani et al., 2011 [24]	1442	ND
Chen et al., 2007 [25]	468	ND
Dart et al., 2007 [26]	7016	ND
Dibra et al., 2003 [27]	1152	CABG, MT
Eisen et al., 2008 [28]	361	ND
Eldrup et al., 2012 [29]	1090	PCI, MT, CABG
Chocron et al., 2008 [30]	2489	ND
Gehi et al., 2008 [31]	929	PCI, MT, CABG
Georgiadou et al., 2010 [32]	101	ND
Glaser et al., 2006 [33]	1457	MT
Harutyunyan et al., 2011 [34]	4372	ND
Hjemdahl et al., 2006 [35]	807	ND
Hueb et al., 2010 [36]	611	MT
Jeremias et al., 2008 [37]	7592	PCI, MT, CABG
Johansen et al., 2006 [38]	507	PCI, MT, CABG
Kaneko et al., 2013 [39]	747	PCI
Ku et al., 2011 [40]	981	ND
Leu et al., 2004 [41]	150	PCI, MT, CABG
Lopes et al., 2008 [42]	825	ND
Máchal et al., 2014 [43]	150	PCI, MT, CABG
Makino et al., 2010 [44]	626	ND
Momiyama et al., 2009 [45]	373	PCI, MT
Muzzarelli and Pfisterer, 2006 [46]	253	PCI, MT, CABG
Papa et al., 2008 [47]	422	NF
Park et al., 2014 [48]	203	PCI, MT, CABG
Pedersen et al., 2010 [49]	1025	PCI, MT, CABG
Povsic et al., 2015 [50]	1908	MT
Roman et al., 2010 [51]	178	PCI, MT, CABG
Rubulis et al., 2010 [52]	187	ND
Sabatine et al., 2007 [53]	3766	ND
Schnabel et al., 2010 [54]	1781	ND
Sinning et al., 2006 [55]	1806	ND
Cihan et al., 2010 [56]	2449	PCI, MT, CABG
Van Melle et al., 2010 [57]	839	PCI, MT
Wakabayashi et al., 2010 [58]	1944	PCI, MT, CABG
Zebrack et al., 2002 [57]	599	ND

## Additional Points

The main highlights of this paper are as follows:

1. We performed a meta-analysis of studies in patients with stable angina to assess which variables predict prognosis.
2. Thirty-eight reports were retained for meta-analysis, representing a total of 101,551 patients.
3. Male sex, reduced EF, diabetes, prior AMI, and high C-reactive protein were the most powerful predictors of cardiovascular events.
4. Metaregression revealed no interaction between the index treatment patients received (PCI, CABG, or OMT) and the incidence of MACE during follow-up.
5. Simple and low-cost clinical features may help clinicians in identifying the most appropriate diagnostic and therapeutic approaches within the broad range of outpatients presenting with stable coronary artery disease.

## References

[1] Lim G. B. (2013). Global burden of cardiovascular disease. *Nature Reviews Cardiology*.

[2] Daly C. A., De Stavola B., Sendon J. L. (2006). Predicting prognosis in stable angina-results from the Euro heart survey of stable angina: prospective observational study. *British Medical Journal*.

[3] Ford E. S., Giles W. H., Croft J. B. (2000). Prevalence of nonfatal coronary heart disease among American adults. *American Heart Journal*.

[4] Ridker P. M., Rifai N., Rose L., Buring J. E., Cook N. R. (2002). Comparison of C-reactive protein and low-density lipoprotein cholesterol levels in the prediction of first cardiovascular events. *The New England Journal of Medicine*.

[5] Wang T. J., Larson M. G., Levy D. (2004). Plasma natriuretic peptide levels and the risk of cardiovascular events and death. *The New England Journal of Medicine*.

[6] Cesari M., Penninx B. W. J. H., Newman A. B. (2003). Inflammatory markers and onset of cardiovascular events: results from the Health ABC study. *Circulation*.

[7] Blankenberg S., McQueen M. J., Smieja M. (2006). Comparative impact of multiple biomarkers and N-terminal pro-brain natriuretic peptide in the context of conventional risk factors for the prediction of recurrent cardiovascular events in the Heart Outcomes Prevention Evaluation (HOPE) study. *Circulation*.

[8] Pijls N. H. J., Fearon W. F., Tonino P. A. L. (2010). Fractional flow reserve versus angiography for guiding percutaneous coronary intervention in patients with multivessel coronary artery disease: 2-year follow-up of the FAME (Fractional Flow Reserve Versus Angiography for Multivessel Evaluation) study. *Journal of the American College of Cardiology*.

[9] Boden W. E., O'Rourke R. A., Teo K. K. (2007). Optimal medical therapy with or without PCI for stable coronary disease. *The New England Journal of Medicine*.

[10] Moher D., Liberati A., Tetzlaff J., Altman D. G. (2009). Preferred reporting items for systematic reviews and meta-analyses: the PRISMA statement. *The British Medical Journal*.

[11] Moher D., Cook D. J., Eastwood S., Olkin I., Rennie D., Stroup D. F. (1999). Improving the quality of reports of meta-analyses of randomised controlled trials: the QUOROM statement. Quality of reporting of meta-analyses. *The Lancet*.

[12] Stroup D. F., Berlin J. A., Morton S. C. (2000). Meta-analysis of observational studies in epidemiology: a proposal for reporting. *The Journal of the American Medical Association*.

[13] Higgins J. P. T., Green S. (2009). *Cochrane Handbook for Systematic Reviews of Interventions Version 5.0.2*.

[14] Biondi-Zoccai G. G. L., Lotrionte M., Abbate A. (2006). Compliance with QUOROM and quality of reporting of overlapping meta-analyses on the role of acetylcysteine in the prevention of contrast associated nephropathy: case study. *The British Medical Journal*.

[15] Higgins J. P. T., Thompson S. G., Deeks J. J., Altman D. G. (2003). Measuring inconsistency in meta-analyses. *British Medical Journal*.

[16] Wilczynski N. L., Haynes R. B., Eady A. (2004). Developing optimal search strategies for detecting clinically sound prognostic studies in MEDLINE: an analytic survey. *BMC Medicine*.

[17] McManus R. J., Wilson S., Delaney B. C. (1998). Review of the usefulness of contacting other experts when conducting a literature search for systematic reviews. *BMJ*.

[18] Aguilar D., Fisher M. R., O'Connor C. M. (2006). Metabolic syndrome, C-reactive protein, and prognosis in patients with established coronary artery disease. *American Heart Journal*.

[19] Arroyo-Espliguero R., Avanzas P., Quiles J., Kaski J. C. (2009). Predictive value of coronary artery stenoses and C-reactive protein levels in patients with stable coronary artery disease. *Atherosclerosis*.

[20] Avanzas P., Arroyo-Espliguero R., Quiles J., Roy D., Kaski J. C. (2005). Elevated serum neopterin predicts future adverse cardiac events in patients with chronic stable angina pectoris. *European Heart Journal*.

[21] Bhatt D. L., Eagle K. A., Ohman E. M. (2010). Comparative determinants of 4-year cardiovascular event rates in stable outpatients at risk of or with atherothrombosis. *The Journal of the American Medical Association*.

[22] Borges J. C., Lopes N., Soares P. R. (2010). Five-year follow-up of angiographic disease progression after medicine, angioplasty, or surgery. *Journal of Cardiothoracic Surgery*.

[23] Breeman A., Bertrand M. E., Ottervanger J. P. (2006). Diabetes does not influence treatment decisions regarding revascularization in patients with stable coronary artery disease. *Diabetes Care*.

[24] Carpeggiani C., Landi P., Michelassi C., Barberini E., L'Abbate A. (2011). Long-term prognosis in stable angina; medical treatment or coronary revascularization in patients younger than 70 years?. *International Journal of Cardiology*.

[25] Chen W.-H., Cheng X., Lee P.-Y. (2007). Aspirin resistance and adverse clinical events in patients with coronary artery disease. *The American Journal of Medicine*.

[26] Dart A. M., Otterstad J. E., Kirwan B.-A. (2007). Predictive value of local and core laboratory echocardiographic assessment of cardiac function in patients with chronic stable angina: the ACTION study. *European Journal of Echocardiography*.

[27] Dibra A., Mehilli J., Braun S. (2003). Association between C-reactive protein levels and subsequent cardiac events among patients with stable angina treated with coronary artery stenting. *The American Journal of Medicine*.

[28] Eisen A., Tenenbaum A., Koren-Morag N. (2008). Calcification of the thoracic aorta as detected by spiral computed tomography among stable angina pectoris patients: association with cardiovascular events and death. *Circulation*.

[29] Eldrup N., Kragelund C., Steffensen R., Nordestgaard B. G. (2012). Prognosis by C-reactive protein and matrix metalloproteinase-9 levels in stable coronary heart disease during 15 years of follow-up. *Nutrition, Metabolism & Cardiovascular Diseases*.

[30] Chocron S., Baillot R., Rouleau J. L. (2008). Impact of previous percutaneous transluminal coronary angioplasty and/or stenting revascularization on outcomes after surgical revascularization: insights from the imagine study. *European Heart Journal*.

[31] Gehi A. K., Ali S., Na B., Schiller N. B., Whooley M. A. (2008). Inducible ischemia and the risk of recurrent cardiovascular events in outpatients with stable coronary heart disease: the heart and soul study. *Archives of Internal Medicine*.

[32] Georgiadou P., Iliodromitis E. K., Kolokathis F. (2010). Osteopontin as a novel prognostic marker in stable ischaemic heart disease: a 3-year follow-up study. *European Journal of Clinical Investigation*.

[33] Glaser R., Selzer F., Jacobs A. K. (2006). Effect of gender on prognosis following percutaneous coronary intervention for stable angina pectoris and acute coronary syndromes. *American Journal of Cardiology*.

[34] Harutyunyan M. J., Mathiasen A. B., Winkel P. (2011). High-sensitivity C-reactive protein and N-terminal pro-B-type natriuretic peptide in patients with stable coronary artery disease: a prognostic study within the CLARICOR Trial. *Scandinavian Journal of Clinical and Laboratory Investigation*.

[35] Hjemdahl P., Eriksson S. V., Held C., Forslund L., Näsman P., Rehnqvist N. (2006). Favourable long term prognosis in stable angina pectoris: an extended follow up of the angina prognosis study in Stockholm (APSIS). *Heart*.

[36] Hueb W., Lopes N., Gersh B. J. (2010). Ten-year follow-up survival of the medicine, angioplasty, or Surgery Study (MASS II): A randomized controlled clinical trial of 3 therapeutic strategies for multivessel coronary artery disease. *Circulation*.

[37] Jeremias A., Kleiman N. S., Nassif D. (2008). Coronary intervention: results from the evaluation of drug eluting stents and ischemic among patients with stable coronary artery disease undergoing percutaneous prevalence and prognostic significance of preprocedural cardiac troponin elevation events registry. *Circulation*.

[38] Johansen A., Høilund-Carlsen P. F., Vach W., Christensen H. W., Møldrup M., Haghfelt T. (2006). Prognostic value of myocardial perfusion imaging in patients with known or suspected stable angina pectoris: evaluation in a setting in which myocardial perfusion imaging did not influence the choice of treatment. *Clinical Physiology and Functional Imaging*.

[39] Kaneko H., Yajima J., Oikawa Y. (2013). Recent characteristics and outcomes of Japanese stable angina pectoris after percutaneous coronary intervention: an observational cohort study using the Shinken database. *International Heart Journal*.

[40] Ku I. A., Farzaneh-Far R., Vittinghoff E., Zhang M. H., Na B., Whooley M. A. (2011). Association of low leptin with cardiovascular events and mortality in patients with stable coronary artery disease: the Heart and Soul study. *Atherosclerosis*.

[41] Leu H.-B., Lin C.-P., Lin W.-T., Wu T.-C., Chen J.-W. (2004). Risk stratification and prognostic implication of plasma biomarkers in nondiabetic patients with stable coronary artery disease. The role of high-sensitivity C-reactive protein. *Chest*.

[42] Lopes N. H., Paulitsch F. D. S., Gois A. F. (2008). Impact of number of vessels disease on outcome of patients with stable coronary artery disease: 5-year follow-up of the Medical, Angioplasty, and bypass Surgery study (MASS). *European Journal of Cardio-Thoracic Surgery*.

[43] Máchal J., Pávková-Goldbergová M., Hlinomaz O., Groch L., Vašků A. (2014). Patients with chronic three-vessel disease in a 15-year follow-up study: genetic and non-genetic predictors of survival. *Medicine*.

[44] Makino A., Nakamura T., Hirano M. (2010). High plasma levels of macrophage migration inhibitory factor are associated with adverse long-term outcome in patients with stable coronary artery disease and impaired glucose tolerance or type 2 diabetes mellitus. *Atherosclerosis*.

[45] Momiyama Y., Kawaguchi A., Kajiwara I. (2009). Prognostic value of plasma high-sensitivity C-reactive protein levels in Japanese patients with stable coronary artery disease: the Japan NCVC-Collaborative Inflammation Cohort (JNIC) Study. *Atherosclerosis*.

[46] Muzzarelli S., Pfisterer M. (2006). Anemia as independent predictor of major events in elderly patients with chronic angina. *American Heart Journal*.

[47] Papa A., Emdin M., Passino C., Michelassi C., Battaglia D., Cocci F. (2008). Predictive value of elevated neutrophil-lymphocyte ratio on cardiac mortality in patients with stable coronary artery disease. *Clinica Chimica Acta*.

[48] Park K.-H., Han S. J. I., Kim H.-S. (2014). Impact of Framingham risk score, flow-mediated dilation, pulse wave velocity, and biomarkers for cardiovascular events in stable angina. *Journal of Korean medical science*.

[49] Pedersen E. R., Ueland T., Seifert R. (2010). Serum osteoprotegerin levels and long-term prognosis in patients with stable angina pectoris. *Atherosclerosis*.

[50] Povsic T. J., Broderick S., Anstrom K. J. (2015). Predictors of long-term clinical endpoints in patients with refractory angina. *Journal of the American Heart Association*.

[51] Roman R. M., Camargo P. V., Borges F. K., Rossini A. P., Polanczyk C. A. (2010). Prognostic value of myeloperoxidase in coronary artery disease: comparison of unstable and stable angina patients. *Coronary Artery Disease*.

[52] Rubulis A., Bergfeldt L., Rydén L., Jensen J. (2010). Prediction of cardiovascular death and myocardial infarction by the QRS-T angle and T vector loop morphology after angioplasty in stable angina pectoris: an 8-year follow-up. *Journal of Electrocardiology*.

[53] Sabatine M. S., Morrow D. A., O'Donoghue M. (2007). Prognostic utility of lipoprotein-associated phospholipase A2 for cardiovascular outcomes in patients with stable coronary artery disease. *Arteriosclerosis, Thrombosis, and Vascular Biology*.

[54] Schnabel R. B., Schulz A., Messow C. M. (2010). Multiple marker approach to risk stratification in patients with stable coronary artery disease. *European Heart Journal*.

[55] Sinning J.-M., Bickel C., Messow C.-M. (2006). Impact of C-reactive protein and fibrinogen on cardiovascular prognosis in patients with stable angina pectoris: the AtheroGene study. *European Heart Journal*.

[56] Cihan S., Onuma Y., Magro M. (2010). Four-year clinical outcome of sirolimus- and paclitaxel-eluting stents compared to bare-metal stents for the percutaneous treatment of stable coronary artery disease. *Catheterization and Cardiovascular Interventions*.

[57] Van Melle J. P., Bot M., De Jonge P., De Boer R. A., Van Veldhuisen D. J., Whooley M. A. (2010). Diabetes, glycemic control, and new-onset heart failure in patients with stable coronary artery disease: data from the heart and soul study. *Diabetes Care*.

[58] Wakabayashi K., Delhaye C., Maluenda G. (2010). Prognosis of asymptomatic coronary artery disease after percutaneous coronary intervention. *American Journal of Cardiology*.

[59] Zebrack J. S., Anderson J. L., Maycock C. A., Horne B. D., Bair T. L., Muhlestein J. B. (2002). Usefulness of high-sensitivity C-reactive protein in predicting long-term risk of death or acute myocardial infarction in patients with unstable or stable angina pectoris or acute myocardial infarction. *American Journal of Cardiology*.

[60] Rapsomaniki E., Shah A., Perel P. (2014). Prognostic models for stable coronary artery disease based on electronic health record cohort of 102 023 patients. *European Heart Journal*.

[61] Shaw L. J., Shaw R. E., Bairey Merz C. N. (2008). Impact of ethnicity and gender differences on angiographic coronary artery disease prevalence and in-hospital mortality in the American College of Cardiology-National Cardiovascular Data Registry. *Circulation*.

[62] Cohn J. N., Rector T. S. (1988). Prognosis of congestive heart failure and predictors of mortality. *The American Journal of Cardiology*.

[63] Velazquez E. J., Francis G. S., Armstrong P. W. (2004). An international perspective on heart failure and left ventricular systolic dysfunction complicating myocardial infarction: the VALIANT registry. *European Heart Journal*.

[64] Task Force Members, Montalescot G., Sechtem U. (2013). 2013 ESC guidelines on the management of stable coronary artery disease. *European Heart Journal*.

[65] Emond M., Mock M. B., Davis K. B. (1994). Long-term survival of medically treated patients in the Coronary Artery Surgery study (CASS) registry. *Circulation*.

[66] Steg P. G., Bhatt D. L., Wilson P. W. (2007). One-year cardiovascular event rates in outpatients with atherothrombosis. *The Journal of the American Medical Association*.

[67] Gulliksson M., Wedel H., Köster M., Svärdsudd K. (2009). Hazard function and secular trends in the risk of recurrent acute myocardial infarction: 30 years of follow-up of more than 775 000 incidents. *Circulation: Cardiovascular Quality and Outcomes*.

[68] Wang H., Eitzman D. T. (2013). Acute myocardial infarction leads to acceleration of atherosclerosis. *Atherosclerosis*.

[69] Kannel W. B., Feinleib M. (1972). Natural history of angina pectoris in the Framingham study. *The American Journal of Cardiology*.

[70] Murabito J. M., Evans J. C., Larson M. G., Levy D. (1993). Prognosis after the onset of coronary heart disease: an investigation of differences in outcome between the sexes according to initial coronary disease presentation. *Circulation*.

[71] Bellasi A., Lacey C., Taylor A. J. (2007). Comparison of prognostic usefulness of coronary artery calcium in men versus women (results from a meta- and pooled analysis estimating all-cause mortality and coronary heart disease death or myocardial infarction). *American Journal of Cardiology*.

[72] Orencia A., Bailey K., Yawn B. P., Kottke T. E. (1993). Effect of gender on long-term outcome of angina pectoris and myocardial infarction/sudden unexpected death. *The Journal of the American Medical Association*.

[73] Huxley R., Barzi F., Woodward M. (2006). Excess risk of fatal coronary heart disease associated with diabetes in men and women: meta-analysis of 37 prospective cohort studies. *BMJ*.

[74] Franco O. H., Steyerberg E. W., Hu F. B., Mackenbach J., Nusselder W. (2007). Associations of diabetes mellitus with total life expectancy and life expectancy with and without cardiovascular disease. *Archives of Internal Medicine*.

[75] Wingard D. L., Barrett-Connor E. L., Ferrara A. (1995). Is insulin really a heart disease risk factor?. *Diabetes Care*.

[76] Cull C. A., Neil H. A. W., Holman R. R. (2004). Changing aspirin use in patients with Type 2 diabetes in the UKPDS. *Diabetic Medicine*.

[77] Tonstad S., Rosvold E. O., Furu K., Skurtveit S. (2004). Undertreatment and overtreatment with statins: the Oslo Health Study 2000-2001. *Journal of Internal Medicine*.

[78] Wild S., Roglic G., Green A., Sicree R., King H. (2004). Global prevalence of diabetes: estimates for the year 2000 and projections for 2030. *Diabetes Care*.

[79] Wilson P. W. F., Nam B.-H., Pencina M., D'Agostino R. B., Benjamin E. J., O'Donnell C. J. (2005). C-reactive protein and risk of cardiovascular disease in men and women from the Framingham Heart study. *Archives of Internal Medicine*.

[80] Folsom A. R., Chambless L. E., Ballantyne C. M. (2006). An assessment of incremental coronary risk prediction using C-reactive protein and other novel risk markers: the Atherosclerosis Risk In Communities study. *Archives of Internal Medicine*.

[81] Danesh J., Wheeler J. G., Hirschfield G. M. (2004). C-reactive protein and other circulating markers of inflammation in the prediction of coronary heart disease. *The New England Journal of Medicine*.

[82] Hemingway H., Philipson P., Chen R. (2010). Evaluating the quality of research into a single prognostic biomarker: a systematic review and metaanalysis of 83 studies of C-reactive protein in stable coronary artery disease. *PLoS Medicine*.

[83] Greenland P., O’Malley P. G. (2005). When is a new prediction marker useful? A consideration of lipoprotein-associated phospholipase A_2_ and C-reactive protein for stroke risk. *Archives of Internal Medicine*.

[84] De Bruyne B., Pijls N. H. J., Kalesan B. (2012). Fractional flow reserve-guided PCI versus medical therapy in stable coronary disease. *New England Journal of Medicine*.

[85] Kip K. E., Hollabaugh K., Marroquin O. C., Williams D. O. (2008). The problem with composite end points in cardiovascular studies. the story of major adverse cardiac events and percutaneous coronary intervention. *Journal of the American College of Cardiology*.

